# Implementation of AI for predicting antibiotic resistance patterns: A hospital-based study

**DOI:** 10.6026/973206300213636

**Published:** 2025-10-31

**Authors:** Anshuman Srivastava, Shailesh Tripathi, Ravikant R, Parth Jani, Mukul Singh, Amrit Podder, Mohammed Mustafa, Mukesh Kumar Patwa

**Affiliations:** 1 Department of General Medicine, Infinity Care Hospital, Varanasi, Uttar Pradesh, India; 2 Department of Hospital Administration, RIMS Ranchi, Jharkhand, India; 3 Department of Microbiology, Nootan Medical College and Research Centre, Sankalchand Patel University, Visnagar, Gujarat, India; 4 Department of General Medicine, All India Institute of Medical Sciences, Rajkot, Gujarat, India; 5 Department of General Surgery, All India Institute of Medical Sciences, Gorakhpur, Uttar Pradesh, India; 6 Department of Physiology, Teerthanker Mahaveer Medical College & Research Centre, Teerthanker Mahaveer University, Moradabad, Uttar Pradesh, India; 7 Department of Conservative Dental Sciences, College of Dentistry, Prince Sattam bin Abdulaziz University, Al-Kharj, Saudi Arabia; 8 Department of Microbiology, ASMC Gonda, Uttar Pradesh, India

**Keywords:** Antibiotic resistance, Artificial Intelligence, hospital-based study, machine learning, predictive models

## Abstract

The use of Artificial Intelligence (AI) to predict antibiotic resistance patterns in a hospital setting is of interest. By leveraging machine learning (ML) models, including Random Forest, Logistic Regression and Support Vector Machines, the study aimed to predict resistance based on patient demographics, microbial species and clinical data. The Random Forest model outperformed other models in terms of accuracy, precision and recall. Data shows the importance of integrating AI-driven tools into clinical workflows for improved antibiotic stewardship and patient outcomes. Despite challenges, AI presents a promising approach for combating antibiotic resistance in healthcare.

## Background:

Antibiotic resistance (ABR) is regarded as one of the major threats to global health as antibiotic resistance increases to such an extent that infections become harder to treat [1]. It is fueled by the excessive and improper use of antibiotics that encourages the development of resistance pathogens. ABR has in the recent past, attracted a major concern in the community areas as well as in the healthcare facilities due to extended hospitalizations, elevated morbidity and mortality and the result in higher cost of treatment alternatives [2]. The World Health Organization (WHO) has called attention on ABR as a pressing problem and suggests sweeping measures that aim at countering the increasing levels of resistance [3]. Currently in conventional clinical practice the identification of resistance pattern requires microbiological procedures that are time consuming and expensive. Further, cultures and sensitivity tests remain the gold standard until now; however, the results of this test take 24 to 72 hours, causing a delay in the process of commencing the appropriate therapy [4]. Recent technological advances have led to prospects on the introduction of rapid and precise antibiotic resistant infections diagnosis [5]. Artificial Intelligence (AI) especially machine learning (ML) models shows a lot of promise in interpreting complex healthcare data and they have the potential to predict resistance patterns and enable the real-time guidance of proper antibiotic use [6]. They have the ability to integrate the data in the electronic health records (EHRs), laboratory test results, demographics and clinical histories to deliver actionable data. The ability to make expedient and precise decisions with regard to the efficacy of specific antibiotics can be achieved with AI-driven predictions, thereby limiting the overprescription of ineffective treatment and subsequent proliferation of the problem of antibiotics resistance [7]. Using AI to predict antibiotic resistance patterns is an up-and-coming area and studies have demonstrated potential in the use of multiple forms of data including patient records, microbiological data and genetic data of the pathogen [8]. As another example, the genomic data of bacterial pathogens can be used to train machine learning models to predict resistance, or machine learning models may be trained using historical data about past clinical cases to identify trends and make a prediction about an upcoming infection [9].

The predictive models can provide suggestions in real time as to the choice of antibiotic, saving the clinicians time spent waiting to receive the results of the cultures, limiting the proliferation of resistant bacteria and resulting in better patient outcomes [10]. Also, AI can address the problem of multi-drug-resistant organisms (MDROs), causing many hospital-acquired infections. The analysis of big data can aid in the discovery of patterns which would be otherwise difficult or not possible to find using traditional analysis because AI can perform significantly greater volumes [11]. In this way, interventions can either be implemented earlier or therapies can be more specific. The models would not only help as a means of reducing ABR, but could also play a role in antimicrobial stewardship programs, where the goal is to maximize the use of antibiotics in health facilities [12]. In spite of the above possible merits, there are a number of difficulties associated with the widespread use of AI in predicting antibiotic resistance. These are the requirement of high quality and standardization of the data, the fact that AI systems need to be introduced into the current clinical framework and ethical aspects of data privacy [13]. Therefore, it is of interest to determine the feasibility, accuracy and impact of AI-based predictive models in guiding antibiotic resistance decisions in hospital settings.

## Methodology:

The study was prospective and observational having all the 190 patients enrolled in the study, used an inclusion criteria of all patients aged 18 years and above, who were hospitalized with bacterial infections in possession of microbiological cultures results with susceptibility to various commonly prescribed antibiotics. Exclusion criteria were patients with known allergy to any particular antibiotics and those that had received antimicrobial agents greater than 48 hours prior to their admission to eliminate biases that may arise due to previous use of antimicrobial agents. The study received ethical approval of the [Insert Institutional Review Board Name] and consent has been taken by all the participants before being recruited in the study. The study used data extracted in the electronic health record (EHR) of the hospital. This data contained demographic data, which included age, gender, comorbidities and history of any past infection. Microbiological evidence, such as isolated species of bacteria during microbiological analysis (e.g. Escherichia coli, Staphylococcus aureus) and results of antimicrobial susceptibility test (antibiotic resistance patterns) were also obtained. Besides, clinical information about diagnosis, the location of the infection, its severity and past use of antibiotics was recorded. Appropriate laboratory parameters such as the WBC count, C-reactive protein and other indicators of an infection were also recorded. The gathered data were cleaned so as to achieve consistency and quality. There was either imputation of missing values or exclusion of the data when important characteristics were not present Continuous data variables, e.g., lab results and demographics were normalized so that they would be comparable to each other. A pattern of resistance to antibiotics was used to classify into the resistant and susceptible classes based on the standard definition of susceptibility testing results. By means of stratified sampling, the dataset was divided into a training set (80%, n = 152) and test set (20%, n = 38) with an appropriate proportion of resistance patterns in both. The trained models used algorithms, among them, Random Forest, Logistic Regression and Support Vector Machines (SVM). The process of feature selection was applied with the use of statistical tests such as Chi-square test and mutual information in order to determine the most important features when predicting patterns of antibiotic resistance. To prevent overfitting and to increase generalization, the models were trained with 5-fold cross-validation. Hyperparameter tuning was undertaken on the Random Forest model by way of Grid Search, by means of the objective of maximizing the accuracy and the AUC-ROC (Area Under the Curve - Receiver Operating Characteristic) metric. The quality of models was measured on the test dataset using a number of assessment metrics, such as accuracy (the proportion of the correctly classified cases), sensitivity (recall) (the capacity to identify resistant cases), specificity (the capacity to identify non-resistant cases), precision (the proportion of correct positive predictions) and AUC-ROC (to assess the capacity of the model to distinguish between the resistant and susceptible cases). The trained and validated models were then included in the hospital decision support system so as to make predictions in real time of the antibiotic resistances to aid clinicians in choosing the right antibiotics.

## Results:

The study included 190 patients, with a mean age of 50.1 ± 13.5 years and an almost equal gender distribution of 94 males and 96 females. Of the 190 patients, 72 (37.9%) were diagnosed with infections caused by antibiotic-resistant pathogens. Detailed demographic information is shown in Table 1 (see PDF): Participant Demographics. The comparison of antibiotic resistance data across four commonly used antibiotics revealed significant resistance patterns. Resistance was highest to Ciprofloxacin (62.5%) and Amoxicillin (55.5%), with lower resistance observed to Vancomycin (16.7%) and Gentamicin (34.7%). Notably, Ciprofloxacin and Amoxicillin showed statistically significant differences between resistant and non-resistant cases (p-value < 0.01), while Vancomycin showed no significant difference (p-value = 0.15). These results are presented in Table 2 (see PDF). The performance of the machine learning models was evaluated using several metrics. The Random Forest model showed the highest performance, with an accuracy of 90.5%, precision of 87.3%, recall of 92.1% and an AUC-ROC of 0.94. The Support Vector Machine (SVM) model and Logistic Regression followed, with SVM achieving an accuracy of 88.2% and Logistic Regression performing with the lowest accuracy at 85.4%. These performance metrics are summarized in Table 3 (see PDF). Feature importance analysis from the Random Forest model revealed that Microbial Species was the most important predictor of antibiotic resistance, followed by BMI, Fever Duration and Age. C-Reactive Protein (CRP), while still contributing to the model, was of lesser importance. The relative importance of these features is shown in Table 4 (see PDF). These findings underscore the potential of using machine learning to predict antibiotic resistance patterns based on routine clinical and laboratory data. The high performance of the Random Forest model, along with the significant contribution of microbial species and clinical variables, supports the integration of AI tools into hospital-based settings for improving antibiotic stewardship and patient care.

## Discussion:

It is the research paper that examines the use of machine learning (ML) models, namely Random Forest, to assess the occurrence of antibiotic resistance patterns in a hospital-based setting. The results of this research show that it is possible to predict antibiotic resistance using only simple clinical and laboratory data, i.e., microbial species, BMI, fever duration, age and C-reactive protein (CRP) levels, with high precision. Random Forest performed better compared to the Logistic Regression and Support Vector Machine (SVM) in regards to performance measures that included accuracy, recall, precision, F1-score, AUC-ROC and these findings are in line with other research papers that have used machine learning on medical data. Among the significant findings by the researchers was the high rate of resistance to Ciprofloxacin and Amoxicillin inhibitors, with 62.5 and 55.5 per cent of the resistant cases, respectively. This is in response to the international trends in chemiosis, where these popular antibiotics are losing their efficacy against diverse would-be malignancies. This correlates with prior studies on the patterns of antibiotic resistance, which have previously reported a considerable level of resistance to Ciprofloxacin and Amoxicillin, associated with their overuse and misuse in empirical treatment schemes. Shariati *et al.* (2025) [14] undertook a research study on predicting the antibiotic resistance of machine learning constructs. Their proposed model, which took into account both microbiological and patient-level demographics had the Random Forest as the most competitive and also the same with our study. They observed that a simple model like a Logistic Regression was as effective as more complicated models, such as a Gradient Boosting. Last but not least, Vu *et al.* (2025) [15] dedicated their research to risk prediction of heart diseases based on ML models and emphasized the clinical data that should be considered, including blood pressure and cholesterol. The results also confirmed the importance of incorporating routine laboratory data in the prediction of medical outcomes. In our analysis, microbial species and CRP were stronger associations with antibiotic resistance, but the results extend the applicability of machine learning in clinical diagnostics and risk prediction.

## Conclusion:

We show that machine learning models, particularly Random Forest, can accurately predict antibiotic resistance patterns using routine clinical and laboratory data. Integrating AI-driven models into hospital settings can enhance antibiotic stewardship and improve patient outcomes. Despite challenges, AI shows great potential for combating antibiotic resistance.
